# Guidelines for the management of people with foot health problems related to rheumatoid arthritis: a survey of their use in podiatry practice

**DOI:** 10.1186/1757-1146-6-23

**Published:** 2013-06-18

**Authors:** Anita E Williams, Andrea S Graham, Samantha Davies, Catherine J Bowen

**Affiliations:** 1 Directorate of Prosthetics, Orthotics and Podiatry, University of Salford, PO29 Brian Blatchford Building, Salford, M6 6PU, UK; 2 Pennine Acute Hospitals NHS Trust, North Manchester General Hospital, Delaunays Rd, Crumpsall, Manchester, M8 5RB, UK; 3 Faculty of Health Sciences, University of Southampton, Building 45, Southampton, SO17 1BJ, UK

**Keywords:** Guidelines, Rheumatoid Arthritis, Foot Health, Podiatry

## Abstract

**Background:**

In the last decade there has been a significant expansion in the body of knowledge on the effects of rheumatoid arthritis (RA) on the foot and the management of these problems. Aligned with this has been the development of specialist clinical roles for podiatrists. However, despite being recommended by national guidelines, specialist podiatrists are scarce. In order to inform non-specialist podiatrists of the appropriate interventions for these foot problems, management guidelines have been developed and disseminated by a group of specialist podiatrists. The aim of this survey was to investigate the use of these guidelines in clinical practice.

**Method:**

Following ethical approval an online questionnaire survey was carried out. The questions were formulated from a focus group and comprised fixed response and open response questions. The survey underwent cognitive testing with two podiatrists before being finalised. An inductive approach using thematic analysis was used with the qualitative data.

**Results:**

245 questionnaires were completed (128–non-specialist working in the private sector, 101 non–specialists working in the NHS and 16 specialist podiatrists). Overall, 97% of the non-specialists (n = 222) had not heard of the guidelines. The non-specialists identified other influences on their management of people with RA, such as their undergraduate training and professional body branch meetings. Three main themes emerged from the qualitative data: (i) the benefits of the foot health management guidelines, (ii) the barriers to the use of guidelines generally and (iii) the features of useable clinical guidelines.

**Conclusions:**

This study has revealed some crucial information about podiatrists’ level of engagement with the foot health management guidelines and the use of guidelines in general. Specifically, the non-specialist podiatrists were less likely to use the foot health management guidelines than the specialist podiatrists. The positive aspects were that for the specialist practitioners, the guidelines helped them to identify their professional development needs and for the few non-specialists that did use them, they enabled appropriate referral to the rheumatology team for foot health management. The barriers to their use included a lack of understanding of the risk associated with managing people with RA and that guidelines can be too long and detailed for use in clinical practice. Suggestions are made for improving the implementation of foot health guidelines.

## Background

In the last decade there has been a significant expansion in the body of knowledge on the effects of rheumatoid arthritis (RA) on the foot. This research has grown from early pioneering work [1,2] and provides evidence for the pathophysiology of foot problems [3-5], the altered biomechanics [6,7], the physical effects [8] and the scale of these problems [9,10]. Further to this, there is now a greater understanding of the impact on the person living with feet affected by RA [11-13]. Foot health management has also been the focus of research that has investigated specific interventions [14,15], the timing of these interventions [16] and the measurement of foot health outcomes [17,18].

Aligned with this increase in evidence and understanding of the impact of RA foot problems, has been the development of specialist clinical roles for podiatrists. As key clinicians involved in the management of foot pathologies, it has been recommended [19-21] that podiatrists are included as core members of the multidisciplinary team alongside consultant rheumatologists, specialist nurses, physiotherapists and occupational therapists. In some secondary care rheumatology units in the UK, specialist podiatrists have expanded their roles through further medical and specialist training to include extended scope practices such as injection therapy, ultrasound imaging [22] and pharmacology. Some roles have evolved that are further specialised with the focus on specific areas of rheumatology, such as foot health management for people who are receiving biologic therapies.

However, there is evidence that a lack of such specialist podiatrists means that there are insufficient numbers to meet the needs of the RA population [23-25] with the results that many people with RA seek foot care from non-specialist podiatrists. These podiatrists have general professional knowledge and skills but have not taken the route to specialisation either through formal training or through what Bacon and Borthwick [26] describe as ‘charismatic authority’. Either route provides the advanced knowledge and skills necessary to manage people with RA, which is not the case for the non-specialists. This is of concern because of the complications associated with the autoimmunity and concomitant drug management, in particular the biologic therapies which may lead to manifestation of infection and/or severe ulceration within the foot and systemic infection [27]. This creates a serious threat to both foot and systemic health. From the patients perspective, they identify the benefits of being managed by specialist podiatrists and report that the seriousness of foot problems can be ignored by those who do not have such a role [12].

The number of specialist podiatrists within rheumatology is unlikely to increase in the current climate within the UK National Health Service (NHS) [28]. However, the need for foot health management remains constant, despite improved medical management of RA [10]. It is known that in the absence of specialist podiatrists, patients will seek foot health management from non-specialists, either within the NHS or in the private sector [12,29]

In order to support podiatrists in their management of people with RA related foot problems, guidelines have been systematically developed by a podiatry led clinical effectiveness group in the NW region of the UK (NWCEG) [30]. These guidelines provide evidence based (and where evidence was lacking, consensus based) standards for foot health management and a screening/referral pathway to guide referrals in cases where foot problems are deteriorating or are impacting general disease management.

The NWCEG guidelines have been widely disseminated throughout the podiatry profession in the UK through undergraduate and postgraduate educational programmes, conference presentations, and publications. However, what was not known was whether the guidelines were being used.

The primary aim of this study therefore, was to investigate podiatrists’ awareness of the NWCEG guidelines, their use of them and the perceived benefits of using them. Further, we aimed to investigate if other RA focussed guidelines [19,21] influenced their practice and what other influences informed their management of people with RA related foot problems. We also aimed to ascertain if there were any barriers to the implementation of guidelines generally and what are considered to be features of usable guidelines within the clinical context. To achieve these aims a survey questionnaire was used to collect both quantitative and qualitative data.

## Method

Following ethical approval from the University of Salford ethics committee, the online questionnaire (Bristol Online Survey http://www.survey.bris.ac.uk/) was designed as a result of a focus group, with non-specialist podiatrists (n = 6), specialist podiatrists (n = 2) and academic colleagues with a specialist interest in rheumatology (n = 2) as participants. The question that triggered the dialogue was, “What do we need to know in order to ensure the effective the use of the NWCEG Guidelines?” The dialogue was digitally recorded and then transcribed verbatim. The transcription was then analysed using a structured framework [31] and the key themes agreed by the participants.

The questions were formulated from themes identified from analysis of the focus group data, with open response questions (qualitative data) [18] and fixed response questions (quantitative data) [n = 4], in order to provide the key features of the participants, such as age, gender and educational level.

The main focus of the questions were in relation to the participant’s knowledge of the currently available guidelines related to management of foot health problems associated with RA (with the focus being the NWCEG guidelines) [30]. The NWCEG guidelines are ‘practitioner facing’ in that they aim to guide the practitioner through the assessment and management aspects of foot care. However, the Arthritis and Musculoskeletal Alliance (ARMA) [19] and the Podiatry Rheumatic Care Association (PRCA) [21] guidelines were also included. The rationale for this was that although these are ‘patient facing’, that is, they aim to define what a person with RA can expect from foot health services, they also contain statements in relation to the podiatrists role in foot health management. In addition, other questions related to whether podiatrists adhered to the guidelines in clinical practice, if there were any other influences on their management of people with RA, what they perceived the benefits of guidelines are and what they considered to be the barriers to their use in clinical practice.

Participants were also asked to identify whether they deemed themselves as either, a specialist podiatrist in rheumatology working within the UK NHS, a non-specialist podiatrist working within the UK NHS or a non-specialist podiatrist working within the UK private sector. Two non-specialist podiatrists completed cognitive testing of the questionnaire. The purpose of this was to check for the clarity of the questions, the positioning of the questions within the questionnaire and the time to complete it (approximately 15 mins). No changes were deemed necessary.

The online survey was promoted through a formal presentation at the UK Society of Chiropodists and Podiatrists annual conference in 2011(attendees N = 1076). The survey was available for the delegates to complete on the computers available at the conference. Additionally, fliers were distributed with the study details and survey link so that if delegates could complete the survey later if they wanted to. The online survey closed six months following the conference.

Quantitative data obtained from the survey questionnaires were analysed using descriptive statistics. An inductive approach using thematic analysis was used with the qualitative data [31] in order to formulate themes. Exemplars from the transcripts were extracted to illuminate these themes. Debate and agreement on the themes was achieved by two of the authors (AW and AG).

## Results

From 245 completed questionnaires, 52.3% (n = 128) were completed by non-specialist podiatrists working within the UK private sector, 41.2% (n = 101) by non-specialist podiatrist working within the UK NHS and 6.5% (n = 16) by specialist podiatrist in rheumatology working within the UK NHS (Table 1).

**Table 1:** Participant demographics

**Total participants (n = 245)**	**Non-specialist private (n = 128)**	**Non-specialist NHS (n = 101)**	Specialist *(n = 16)
Gender	99 female	76 female	10 female
	29 male	25 male	6 male
Years qualified	1-35 (SD = 7.78)	1-29 (SD = 8.71)	6-29 (SD = 6.63)
Qualification:			
Diploma	29	10	0
BSc (hons)	95	89	6
MSc	4	2	8
PhD	0	0	2
Numbers of people with RA managed each week	1-10 (SD = 2.48)	5-28 (SD = 8.02)	15- 45 (SD = 9.34)

*3 with additional academic posts at universities; 1 full time academic.

Overall, the majority of the ‘non-specialist’ podiatrists responding to the survey indicated that they had not heard of the national guidelines. With 99.1% (n = 227) reporting that they had not heard of the ARMA guidelines [19], similarly 96.5% (n = 221) had not heard of the PRCA guidelines [21], and 96.9%, (n = 222) had not heard of the NWCEG guidelines [30] (Table 2).

**Table 2:** Participants Knowledge of Guidelines

**Guideline**	**Response**	**Non-specialist private (n = 128)**	**Non-specialist NHS (n = 101)**	**Specialist NHS (n = 16)**
NW CEG	never heard	120	51	0
Guidelines [30]	read them but not acting on recommendations	6	45	0
	fulfilling recommendations	2	5	16
Arthritis and Musculoskeletal Alliance (Inflammatory Arthritis) [19]	never heard	127	100	0
	read them but not acting on recommendations	1	1	1
	fulfilling recommendations	0	0	15
Musculoskeletal Foot Health Standards [21]	never heard	123	98	0
	read them but not acting on recommendations	5	2	1
	fulfilling recommendations	0	1	15

When asked if guidelines influence their clinical practice in managing patients with rheumatoid arthritis (Table 3), all of the non-specialist podiatrists identified undergraduate education as being the main influence with the more specialist activities such as conferences, training courses and specific web-based information being accessed more by the specialist podiatrists. The influence of guidelines was one of the least mentioned and when they were, the majority identified the National Institute for Clinical Excellence guidelines [20] as being the only influence.

**Table 3:** Most significant influences on clinical practice in relation to managing patients with rheumatoid arthritis (participants were asked to tick all those that applied to them)

	**Non-specialist private (n = 128)**	**Non-specialist NHS (n = 101)**	**Specialist NHS (n = 16)**
Undergraduate education	128	101	16
Local Society of Chiropodists and Podiatrists branch meetings	101	55	2
Reading scientific papers in peer reviewed journals	34	30	16
Guidelines	16	51	16
Conferences	15	56	15
Web based resources e.g. Arthritis Research UK	10	25	1
Informal contact with those specialising in the field	5	12	0
Training courses (BSR Foot and Ankle Course)	0	2	13

Following analysis, the qualitative data was organised into three main themes.

### Theme 1 - The benefits of the NWCEG foot health management guidelines

Of those podiatrists that indicated that they were fulfilling the recommendations, the vast majority were those in specialist posts and their level of use was reported to be high. In relation to the benefits of using the NWCEG guidelines, the specialist podiatrists (S-NHS) indicated that they had impact on the quality of patient care through ensuring that practice was based on evidence;

“Although I specialise in this area I now feel secure in that I am doing the best for my patients in relation to applying the best evidence to my practice”. S-NHS14 (age-35; gender-female; highest educational level-MSc).

And further to this, they support defensible practice;

“…With these I know that I am practicing in the most defensible way.... I can prove that I am practicing to the standard expected based on research evidence”. S-NHS5 (age-40; gender-male; highest educational level-MSc).

The NWCEG guidelines also improved the specialist podiatrists’ confidence in being able to maintain services for these patients;

“…The guidelines mean that I can defend continuing this service to my manager. Rheumatology always comes second to diabetes and these help to maintain a high profile”. S-NHS3 (age-35; gender-female; highest educational level- BSc (hons)).

In addition to their direct management of patients, the guidelines also helped them to identify their Continuing Professional Development needs;

“… I hadn’t thought about using steroid injections before until I saw their use in the guidelines …I have done the training and use it in practice now”. S-NHS4 (age-34; gender-female; highest educational level-BSc(hons)).

For the 5 non-specialist NHS and the 2 non-specialist private podiatrists who reported that they were using the NWCEG guidelines, the benefits are perceived to be different to the specialist podiatrists. They recognised that the guideline screening and referral pathway had helped them to ensure that the patients were being managed in the right location;

“.....helped me to identify those patients that I can’t manage as I don’t work within a rheumatology team”. NS-NHS5 (age-29; gender-female; highest educational level-BSc (hons)).

“…some of the patients are best managed in the rheumatology department…those on the new drugs and those that need foot surgery or footwear”. NS-P1 (age-42; gender-female; highest educational level-BSc (hons)).

Further, the key standards had supported the implementation of aspects of management that they had learned about during their undergraduate training,

“I am working on maintaining these standards and use them as reference to support what I learned at uni…. I would not have done this without the standards”. NS-NHS2 (age-24; gender- male; highest educational level- BSc (hons)).

“…the key messages help me to identify the ‘must do’s…I did know about some of these but it’s hard to remember all from training”. NS-P2 (age-28; gender-female; highest educational level-BSc (hons)).

The benefits to the specialist podiatrists are clear in that they have been used to support good quality patient care such as role development, maintaining services, defensible practice and applying evidence into practice.

From the few who are fulfilling the standards in non-specialist posts, the NWCEG guidelines had provided guidance as to the most appropriate location of management and as an aide memoir to aspects of management that had been forgotten since training. Overall, by those who knew about them, the management guidelines were identified as being useful in the context of direct and indirect aspects of patient management.

### Theme 2 - Barriers to the use of guidelines generally

Non-specialist podiatrists identified that they lacked the time in clinical practice to read any guidelines. Further, they identified that even if guidelines were read, there was little point to them as the standards could not be met due to lack of resources and lack of funding for professional development. Some of the private podiatrists preferred to spend the time researching their own sources of information and making their own decisions.

“I prefer to research and make my own decisions- I am an autonomous practitioner and guidelines don’t allow for clinical judgement”. NS-P20 (age-54; gender-male; highest educational level-BSc (hons)).

“I don’t use them...do not agree with the use of guidelines, they interfere with my autonomy - they prevent me being able to make clinical judgements for each patient…I don’t think my patients would have confidence in me if they knew I used them”. NS-P30 (age-55; gender-male; highest educational level-BSc (hons)).

A number of the private podiatrists thought that guidelines were not relevant to their practice;

“Guidelines are something that don’t really apply to me in my practice as I focus on basic treatments”. NS-P35 (age-45; gender-female; highest educational level- BSc (hons)).

The non-specialist NHS podiatrists reported that there were just too many guidelines and there were issues in the way that guidelines are laid out;

“…there are too many guidelines from different agencies and overlap in information”. NS-NHS78 (age-34; gender-female; highest educational level- BSc (hons)).

“They are too long to read and it’s hard to navigate around what is important and what is supporting information….also they are not that accessible”. NS-NHS54 (age-58; gender- female; highest educational level- diploma).

The specialist podiatrists focussed on concerns about potential conflict in professional roles for interventions contained in guidelines such as steroid injections, rather than the layout and content.

### Theme 3 - The features of useable clinical guidelines

There was agreement across all three participant groups that referral pathways were a useful clinical tool. However, it was thought that guidelines need to be updated on a regular basis and old ones removed from web sites and clinics. Many of the non-specialist NHS group mentioned that diagrams and mapping against clinical practice were useful;

“Diagrams are helpful to understand key concepts such as correction of rear foot with foot orthoses”. NS-NHS44 (age-40; gender-male; highest educational level-BSc (hons)).

“They need to be in a logical sequence…procedures need to reflect what goes on in clinical practice”. NS-NHS56 (age-25; gender-female; highest educational level- BSc (hons)).

With summaries and key points being helpful:

“Summary statements are good…key points of essentials with reference back to the main section for more detail”. NS-NHS34 (age-46; gender-male; highest educational level-MSc).

In relation to the content of guidelines, additional information was suggested such as;

“How to proceed if the patient falls outside of the parameters of the guidelines”. NS-P70 (age-35; gender-female; highest educational level- BSc (hons)).

“Resource links for patient information and lists of courses where you can get training”. NS-NHS22 (age-29; gender- male; highest educational level-BSc (hons)).

“… A way of auditing the standards to ensure that they are being adhered to and then if not it provides a case for service development”. NS-NHS1(age-42; gender-female; highest educational level- BSc (hons)).

One participant suggested that a summary of other relevant guidelines should be contained in each guideline and each identified as to whether they are useful for managers / clinical leads, non-specialists, specialists and/or patients.

## Discussion

This study has revealed some crucial aspects about podiatrists’ engagement in guidelines of relevance to the management of people who present with foot problems related to RA, in particular the NWCEG guidelines [30]. It has demonstrated that, in relation to both the knowledge of and use of RA guidelines there is a notable difference in that the UK specialist podiatrists are far more likely to use the guidelines than UK non-specialist podiatrists. This is of concern as the NWCEG guidelines were intended for all podiatrists to ensure the appropriate and timely management of RA related foot problems.

Additionally, there were differences in responses in relation to barriers to the implementation of guidelines into clinical practice, with the non-specialist podiatrists more frequently reporting difficulties in interpreting guidelines (cognitive barriers) and had less favourable opinions about guidelines (affective barriers) than specialist podiatrists.

The few non-specialists recognising benefits commented more on how they support appropriate referrals to the rheumatology team for foot health management, rather than guiding them through their own management of the patient. However, this is beneficial in relation to the patient receiving the right intervention in the right setting. A few did identify that adhering to the guidelines supported defensible practice but it is of concern that some thought they were not relevant to their practice as their treatment of people with RA was very simple, such as toe nail cutting. This perhaps indicates a lack of knowledge about the implications of even simple foot care for those patients who are immunologically suppressed and/or receiving biological therapy for their systemic disease, and in whom skin and soft tissue infections occur more frequently and can develop rapidly [27]. Indeed the non-specialist podiatrists were less likely to have undertaken postgraduate qualifications in this area.

Some non-specialist podiatrists considered that the guidelines detracted from their professional autonomy and hence they did not use them. Nancarrow and Borthwick [32] have proposed that perceptions such as these arise from professional isolation and may be linked to avoidance of medical hierarchies. This may indicate that, for those podiatrists, their practice is not defendable in terms of new paradigms of management of people with early RA disease [16], as advocated within the guidelines. As such, the ‘window of opportunity’ to ensure early detection and management of foot problems for these patients may be missed.

In contrast, to the non-specialist podiatrists, the specialist podiatrists were using the guidelines. However, they were hampered by external barriers such as a lack of agreement about their roles and responsibilities within rheumatology, particularly in relation to interventions that have traditionally been carried out by the medical profession. This is consistent with Redmond et al. [24] who identified wide variation in the UK in the provision of foot health services and training for specialist podiatry rheumatology services.

A positive perspective from our study was that the ‘specialist’ podiatrists stated that guidelines helped them to identify their professional development needs, specifically in relation to advanced skills and also helped them provide evidence for the provision of a specialist foot health service for people who have RA. A further development from this would be the embedding of foot health care algorithms in clinical practice as well as the design and implementation of an audit tool based on the foot health guidelines in order to formally evaluate services.

In relation to usability of guidelines, there were some comments by the specialist podiatrists as to how this could be improved. Solutions to the cognitive barriers may be simple in relation to the presentation and format of the guidelines. The specifics that were suggested were having a summary of the key aspects of the management guidelines in a separate document and also a summary of all relevant guidelines with an indication as to who they are relevant to (managers, patients, podiatrists). Also, it was suggested that a way of auditing the standards would be useful in order to identify gaps in training and service provision.

Dodek et al. [33] identified the influences on the implementation of guidelines as being the quality of evidence and the credibility of the guidelines development group. However, these were not identified as a barrier in this survey. One of the contextual factors that seem to be implicit in the results of this survey is the influence of the type of service. Dodek et al. [33] further identified that shared beliefs about guidelines and adherence to guideline recommendations may be more evident within teams. Therefore, one of the ways to improve the use of guidelines is to ensure peer support where non-specialist podiatrists are working in isolation. A ‘peer support and review scheme’ as recommended by Piper et al. [34] may help to support links between the specialist and non-specialist services. Further, a service that provides seamless care between specialist and non-specialist services could provide opportunity for support and education [35]. Lineker and Husted [36] concluded that it is difficult to change behavior and noted that recent graduates may be more receptive to guideline implementation. Therefore, it would be pertinent to reinforce the benefits of using the guidelines during the undergraduate training of podiatrists.

There are some limitations to this study in that it was delivered at the UK Society of Chiropodists and Podiatrist’s annual conference and so may not reflect the opinions of all podiatrists practising within the UK. Further, there may be potential bias in the survey such as acquiescent responses, particularly from the specialist podiatrists. It was also impossible to ensure that the survey was not completed more than once by each participant or that a non-podiatrist could have completed it. Also it was impossible to ensure that it was completed by equal numbers of private, non-specialist NHS podiatrists and specialist podiatrists and so it was a pragmatic and convenient sample. However, the proportions of non-specialist (93.5%) to specialist (6.5%) podiatrists who completed the survey reflect the national profile as identified by Redmond et al. [24].

## Conclusion

Within this study we have identified an extremely high percentage of non-specialist podiatrists who are unaware of the guidelines for the management of foot health problems for people who have rheumatoid arthritis. Therefore, implementation strategies need to be improved. Contextual factors, such as peer support, audit and education may support the implementation of the guidelines into non-specialist podiatry practice.

### Consent

Information about consent was provided in the participant information sheet which they read before completing the survey: ‘By completing the survey you are providing consent to be part of this research and for the publication of the results’.

The survey and/or a copy of the NWCEG Guidelines for the Management of Foot Health for People with Rheumatoid Arthritis can be obtained from the lead author a.e.williams1@salford.ac.uk

## Competing interests

The authors declare that they have no competing interests.

## Authors’ contributions

AW conceived of the study and led the development of the survey, AG contributed to the development of the survey and qualitative data analysis, SD contributed to the development of the survey and CB participated in its dissemination and helped to draft the manuscript. All authors read and approved the final manuscript.
